# Risk factors for psychiatric disorders following traumatic brain injury: a multivariate logistic regression analysis

**DOI:** 10.3389/fpsyt.2024.1499894

**Published:** 2024-11-27

**Authors:** Hanyu Liu, Tongjun Yi

**Affiliations:** Huludao Central Hospital, Huludao, Liaoning, China

**Keywords:** traumatic brain injury, psychiatric disorders, unemployment, adaptation, risk factor

## Abstract

**Objective:**

This study aimed to investigate the incidence and risk factors of psychiatric disorders following traumatic brain injury (TBI).

**Methods:**

A total of 232 patients with closed TBI admitted to our hospital from January 2021 to January 2023 were included. Basic demographic data, injury circumstances, and psychiatric conditions during hospitalization were collected. Patients were followed up at 9 months post-injury, and based on clinical interviews, symptoms, and questionnaires, they were categorized into those with post-TBI psychiatric disorders and those without. The study aimed to explore the predictive factors for psychiatric disorders after TBI.

**Results:**

Among the 232 patients, 104 developed psychiatric disorders by the end of the 9-month follow-up, resulting in an incidence rate of 44.83%. The employment rate was significantly lower in the psychiatric disorder group compared to the non-psychiatric disorder group. Additionally, the GCS scores upon admission were significantly higher in the psychiatric disorder group, along with a greater proportion of limb injuries, post-traumatic coma, intracranial hematomas, and frontal lobe injuries. The results of the multivariate logistic regression analysis indicated that unemployment (caused by poor recovery from TBI), lower GCS scores at admission, limb injuries, post-traumatic coma, frontal lobe injuries, and the presence of psychiatric symptoms during hospitalization were independent predictors of psychiatric disorders following TBI.

**Conclusion:**

Unemployment, lower GCS score on admission, limb injury, post-traumatic coma, frontal lobe injury, onset of psychiatric symptoms during hospitalization was identified as independent predictors of post-traumatic psychiatric disorders. Routine mental health screenings for conditions such as depression and anxiety should be integrated into the care of TBI patients.

## 1 Introduction

Traumatic brain injury (TBI) represents a critical global health challenge, with an estimated incidence of approximately 73 cases per 100,000 individuals experiencing severe TBI annually worldwide (1). The risk of TBI is particularly pronounced among males and specific age groups, notably the young and elderly. The principal causes of TBI differ based on geographic and economic contexts, with falls and road traffic accidents being the predominant etiologies. In high-income countries, falls have emerged as the leading cause, especially among older adults, while road traffic accidents remain the primary cause in low- and middle-income nations (2, 3).

Psychiatric disorders constitute significant short-term and long-term sequelae of TBI, affecting approximately 45.9% to 75.48% of patient’s post-injury, a condition referred to as post-traumatic psychiatric disorder (4, 5). Common manifestations include depression, anxiety, psychosis, and personality changes, with major depressive disorder (30.3%) and personality alterations (33.3%) being the most frequently observed types (6). Mental disorders affect patient recovery in many ways, and early detection and intervention are very important (7).

The onset of post-traumatic psychiatric disorders can vary significantly based on pre-existing medical conditions; some disorders may present acutely, while others may develop over time (8). Longitudinal studies indicate that TBI can confer a prolonged risk for developing mental health disorders, with heightened susceptibility to a range of psychiatric conditions, including depression, delusional disorder, and personality disorders (9). Evidence suggests that the occurrence of post-traumatic psychiatric disorders is closely linked to TBI severity, Glasgow Coma Scale scores, injury characteristics, and psychiatric history (10, 11). Given their high incidence and potential for long-term impact, early identification, prevention, and intervention are crucial. The findings of Lindekilde et al., demonstrating the increased risk of type 2 diabetes among individuals with psychiatric disorders, highlight the complex bidirectional relationships between psychiatric and physical health conditions, reinforcing the need for integrative care approaches in managing post-TBI psychiatric outcomes (12).

## 2 Methods

### 2.1 Study design

This study employed a prospective cohort design to investigate the risk factors associated with psychiatric disorders following TBI. A total of 232 patients with closed TBI admitted to our institution between January 2021 and January 2023 were included. All participants underwent a 9-month follow-up post-injury to assess the incidence of psychiatric disorders and identify relevant risk factors. Ethical approval for this research was granted by the institutional review board.

### 2.2 Inclusion and exclusion criteria

#### 2.2.1 Inclusion criteria

1. Patients aged 18 to 75 years, regardless of gender, with a documented history of trauma.
2. Diagnosis of either open or closed TBI confirmed through clinical and radiological evaluations.
3. Acute TBI, defined as a time interval of less than 24 hours between injury and hospital admission.
4. Informed consent was obtained from both patients and their families regarding participation in the study.

#### 2.2.2 Exclusion criteria

1. A history of previous psychiatric disorders or cognitive impairments.
2. Coexisting neurological conditions unrelated to TBI, such as multiple sclerosis or epilepsy.
3. A history of substance abuse or drug dependence.
4. Congenital intellectual disabilities or similar conditions.

### 2.3 Diagnosis of post-traumatic psychiatric disorders

Patients were followed for 9 months post-injury, with outpatient evaluations conducted to determine the presence of psychiatric disorders. The diagnosis of post-traumatic psychiatric disorders was established using structured clinical interviews, the DSM-IV standardized diagnostic criteria, and validated assessment instruments (BDI-II, PHQ-8, PCL-C, MPAI).

#### 2.3.1 Structured clinical interview

This type of interview ensures a systematic assessment of the patient’s symptoms. Based on a series of standardized questions, clinicians gain insight into the patient’s medical history, symptoms, and impact on daily life. This approach can provide both quantitative and qualitative information that can help confirm the diagnosis.

#### 2.3.2 DSM-IV criteria

The diagnosis of mental disorders usually relies on the criteria in the Diagnostic and Statistical Manual of Mental Disorders, Fourth Edition (DSM-IV). Clinicians will match the patient’s symptoms with the specific diagnostic criteria listed in the DSM-IV to determine whether the diagnostic criteria for a psychiatric disorder are met.

#### 2.3.3 Validated assessment scale

- BDI-II (Beck Depression Rating Scale, 2nd Edition): Used to assess the severity of depressive symptoms and help determine whether a patient has a depressive disorder.
- PHQ-8 (Patient Health Questionnaire-8): This is a self-administered questionnaire used to screen for depression and assess emotional state over the past two weeks.
- PCL-C (Post-Traumatic Stress Symptom Scale): Used to assess symptoms of post-traumatic stress disorder (PTSD) and help determine whether a patient is experiencing a post-traumatic psychological response.
- MPAI (Post-Brain Injury Assessment Scale): Used to assess the functional status of patients after brain injury, including cognitive, emotional, and behavioral changes.

By combining these approaches, clinicians can conduct a comprehensive assessment of possible psychiatric disorders following TBI, ensuring an accurate diagnosis and developing an appropriate treatment plan. In practice, the diagnostic process needs to take into account the patient’s specific situation, symptoms, and assessment results.

### 2.4 Influencing factors

#### 2.4.1 Demographic data

This included age, gender, educational attainment, employment status (unemployment caused by poor recovery from TBI), comorbidities, smoking history, and alcohol consumption.

#### 2.4.2 Trauma-related indicators

Factors such as Glasgow Coma Scale (GCS) score upon admission, injury type (high-energy vs. low-energy), time from injury to admission, presence of limb injuries, organ injuries, coma, skull fractures, intracranial hematomas, intracerebral hematomas, frontal lobe injuries, hypoxemia, shock, and seizures during hospitalization were recorded.

#### 2.4.3 Physical and psychiatric symptoms during hospitalization

The occurrence of insomnia, dizziness, headaches, nausea, vomiting, memory impairments, emotional disturbances, and behavioral issues during the hospital stay was documented.

### 2.5 Statistical methods

Statistical analyses were conducted using SPSS version 22.0. Continuous variables were expressed as means ± standard deviations, and intergroup differences were assessed using t-tests. Categorical variables were expressed as proportions, with differences analyzed using chi-square tests. For factors that demonstrated statistically significant differences, multivariate logistic regression analysis was performed. A p-value of <0.05 was considered statistically significant.

## 3 Results

### 3.1 Demographic characteristics

Among the 232 patients, 104 developed psychiatric disorders after a 9-month follow-up, resulting in an incidence rate of 44.83% for post-traumatic psychiatric disorders. Basic demographic data were collected and compared between the two groups, as shown in **Table 1**. No significant differences were found between the groups in terms of age, gender, education level, smoking history, or alcohol consumption (all p > 0.05). However, the unemployed caused by TBI in the psychiatric disorder group was significantly lower than that in the non-psychiatric disorder group (p < 0.05).

**Table 1:** Comparison of demographic characteristics.

Indicator	Mental Disorder	No Mental Disorder	χ²/t-value	*P*-value
n	104	128		
Gender (Male/Female)			0.554	0.457
Male	60	80		
Female	44	48		
Age ()	45.2 ± 12.3	42.8 ± 11.7	1.518	0.138
Range	26-55	27-56		
Mean ± SD	45.2 ± 12.3	42.8 ± 11.7	1.518	0.138
Education Level			3.326	0.190
Primary	20	15		
Secondary	50	60		
Tertiary	34	53		
Employment Status			13.28	<0.001
Unemployed caused by TBI	64	48		
Others	40	80		
Marriage			0.806	0.369
Yes	92	108		
No	12	20		
Hypertension			1.004	0.316
Yes	54	58		
No	50	70		
Coronary Heart Disease			1.579	0.209
Yes	39	38		
No	65	90		
Diabetes			1.237	0.266
Yes	49	51		
No	55	77		
Hyperlipidemia			1.579	0.209
Yes	41	61		
No	63	67		
Smoking History			0.585	0.444
Yes	50	68		
No	54	60		
Alcohol History			1.321	0.250
Yes	40	40		
No	64	88		

### 3.2 Trauma-related factors

Trauma-related factors were collected and compared between the two groups, with results presented in **Table 2**. No significant differences were observed in causes of injury, time from injury to admission, organ injuries, skull fractures, hypoxemia, shock, or seizures during hospitalization (all p > 0.05). The GCS scores upon admission were significantly lower in the psychiatric disorder group, while the proportions of limb injuries, post-traumatic coma, intracranial hematomas, and frontal lobe injuries were also significantly greater in this group compared to the non-psychiatric disorder group (all p < 0.05).

**Table 2:** Comparison of trauma-related factors.

Indicator	Mental Disorder	No Mental Disorder	χ²/t-value	*P*-value
n	104	128		
GCS at Admission (Mean ± SD)	10.52 ± 3.21	12.11 ± 2.83	4.007	<0.001
Cause of Injury			0.210	0.647
High-energy damage	60	70		
Low-energy damage	44	58		
Time from Injury to Admission			0.164	0.921
<6 hour	30	40		
6-12 hours	50	60		
12-24 hours	24	28		
Limb trauma			4.503	0.034
Yes	31	23		
No	73	105		
Organ damage			3.789	0.052
Yes	26	19		
No	78	109		
Post-traumatic coma			12.40	<0.001
Yes	36	19		
No	68	109		
Skull fracture			3.346	0.067
Yes	32	26		
No	72	102		
Intracranial hematoma			4.621	0.032
Yes	21	13		
No	83	115		
Frontal lobe injury			5.927	0.015
Yes	32	22		
No	72	106		
Hypoxemia			1.542	0.214
Yes	21	18		
No	83	110		
Shock			2.573	0.109
Yes	16	11		
No	88	117		
Seizures during hospitalization			2.170	0.141
Yes	10	6		
No	94	122		

### 3.3 Physical and psychiatric symptoms during hospitalization

Physical and psychiatric symptoms during hospitalization were collected and compared, with results shown in **Table 3**. There were no significant differences in the occurrence of dizziness, headaches, nausea, vomiting, memory impairments, or behavioral disturbances between the groups. However, the rates of insomnia and emotional disturbances were significantly higher in the psychiatric disorder group compared to the non-psychiatric disorder group (all p < 0.05).

**Table 3:** Comparison of physical and psychiatric symptoms during hospitalization.

Indicator	Mental Disorder	No Mental Disorder	t-value	*P*-value
n	104	128		
Insomnia			4.069	0.044
Yes	36	29		
No	68	99		
Dizziness and headache			2.906	0.088
Yes	42	38		
No	62	90		
Nausea and vomiting			3.786	0.052
Yes	26	19		
No	78	109		
Memory impairment			3.306	0.069
Yes	16	10		
No	88	118		
Mood disorders			13.27	<0.001
Yes	30	13		
No	74	115		
Behavioral disorders			2.931	0.087
Yes	11	6		
No	93	122		

### 3.4 Multivariate logistic regression analysis of post-traumatic psychiatric Disorders

**Figure 1:**
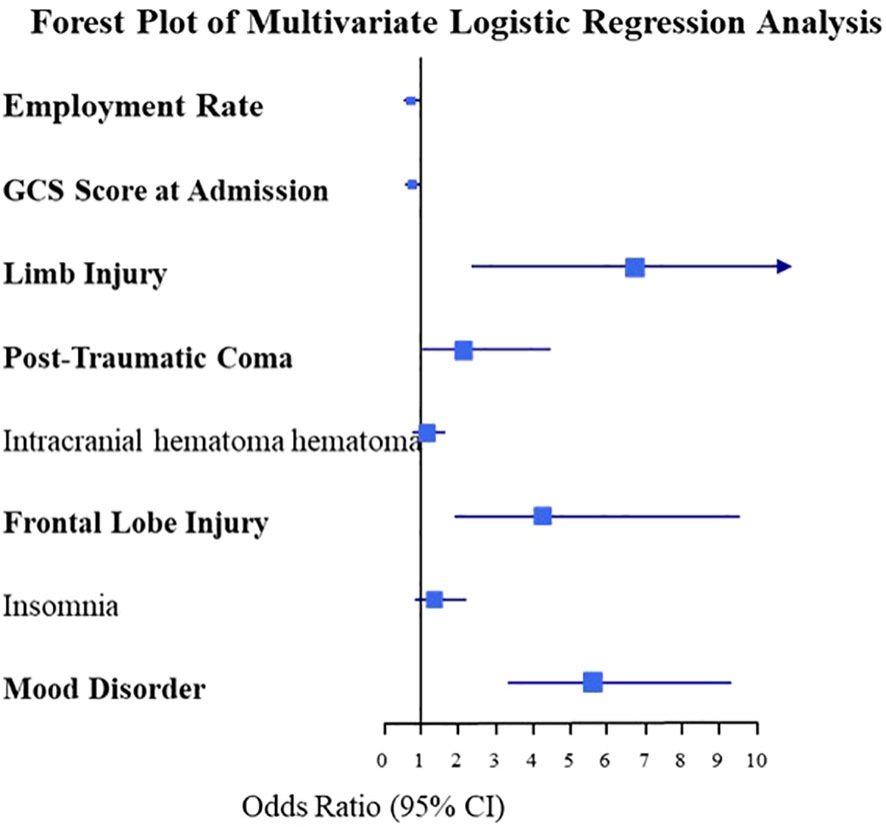
Forest plot of multivariate logistic regression analysis of post-traumatic psychiatric disorders.

Factors exhibiting significant intergroup differences, including employment status, GCS scores upon admission, limb injuries, post-traumatic coma, intracranial hematomas, frontal lobe injuries, insomnia, and emotional disturbances, were subjected to multivariate logistic regression analysis. The results are presented in **Table 4**. The multivariate logistic regression analysis indicated that unemployment, lower GCS scores upon admission, limb injuries, post-traumatic coma, frontal lobe injuries, and the presence of psychiatric symptoms during hospitalization were independent predictors of post-traumatic psychiatric disorders (all p < 0.05). To provide a more intuitive representation of the results, a forest plot was generated, as shown in **Figure 1**.

**Table 4:** Multivariate logistic regression analysis of post-traumatic psychiatric disorders.

	SE	β	OR	95%CI	*P*
Employment Rate	0.21	-0.80	0.45	0.30, 0.68	<0.05
GCS Score at Admission	0.12	-0.29	0.75	0.59, 0.95	<0.05
Limb Injury	0.53	1.91	6.72	2.38, 18.99	<0.05
Post-Traumatic Coma	0.37	0.77	2.15	1.04, 4.44	<0.05
Intracranial hematoma	0.18	0.15	1.16	0.82, 1.65	>0.05
Frontal Lobe Injury	0.41	1.45	4.26	1.91, 9.51	<0.05
Insomnia	0.24	0.31	1.36	0.85, 2.18	>0.05
Mood Disorder	0.26	1.72	5.58	3.35, 9.29	<0.05

## 4 Discussion

TBI represents a significant public health challenge, with consequences that extend beyond physical impairment. Post-traumatic psychiatric disorders frequently occur as complications, affecting a substantial proportion of survivors (13). This study followed 232 TBI patients over a 9-month period to identify risk factors associated with the development of psychiatric disorders post-TBI. Our results indicated that unemployment, lower GCS scores at admission, limb injuries, post-traumatic coma, frontal lobe injuries, and the presence of psychiatric symptoms during hospitalization served as independent predictors of post-traumatic psychiatric disorders. The findings by Cheng et al. on the impact of COVID-19 on patients with psychiatric disorders underscore the importance of recognizing comorbid conditions and tailored care approaches, paralleling the need for targeted interventions in post-TBI psychiatric disorders to mitigate adverse outcomes (14).

We found that unemployed caused by TBI individuals were at an increased risk for developing psychiatric disorders following TBI, which aligns with existing literature highlighting the importance of socioeconomic factors in the risk of mental illness after TBI (9, 15). Unemployment nearly doubled the likelihood of psychiatric disorders compared to employed individuals. Employment not only provides financial stability but also offers essential social support and a sense of purpose, both critical for mental well-being (16). In addition, unemployment can result in reduced social interactions and support, leading to feelings of isolation. Social support is crucial for recovery from traumatic experiences, and a lack of it can hinder resilience and coping mechanisms (1) (17). GCS scores at admission are pivotal in determining injury severity; lower GCS scores are associated with more severe injuries and a heightened risk of psychiatric disorders (18). Som1studies suggest a complex interplay between TBI severity and psychiatric outcomes, indicating that even mild TBIs can increase the risk of mental health issues (9). A meta-analysis of 57 studies found a significant association between prior TBI, including mild cases, and subsequent neurological and psychiatric diagnoses (19). Although the specific mechanisms remain unclear, chronic inflammation following TBI may play a role in the emergence of neuropsychiatric symptoms (20).

Alway et al. (21) identified limb injuries as strong predictors of psychiatric disorders following TBI, likely due to associated pain and disability that can negatively impact quality of life (4). Furthermore, post-traumatic coma was recognized as an independent risk factor for psychiatric disorders. Research indicates that patients experiencing prolonged coma or altered consciousness face a higher risk of developing various mental illnesses, with the severity and duration of coma influencing brain recovery and healing capacity, potentially leading to psychiatric complications (22).

Frontal lobe injuries emerged as independent risk factors for psychiatric disorders. The relationship between frontal lobe damage and psychiatric illness is intricate, as this region is crucial for cognitive functions, emotional regulation, impulse control, and social behavior. Injury to the frontal lobe increases vulnerability to psychiatric disorders (23). The findings by Menkü et al. underscore the diagnostic complexity of psychiatric disorders, emphasizing the potential for misdiagnosis due to underlying medical conditions, which aligns with the need for comprehensive evaluations in patients presenting with post-TBI psychiatric symptoms (24). Studies indicate that TBI significantly heightens the risk of developing new psychiatric conditions, particularly when the frontal lobe is involved (25). The findings of Minen et al. on the association between migraines and psychiatric comorbidities underscore the complex neurocircuitry linking neurological and psychiatric disorders, mirroring the multifaceted risk factors observed in post-TBI psychiatric outcomes (26). Specific regions of the frontal lobe correlate with distinct psychiatric symptoms; for instance, abnormalities in the left and bilateral frontal lobes are linked to depression and schizophrenia, while right frontal lobe abnormalities are associated with mania, and orbitofrontal dysfunction relates to obsessive behaviors (27). The findings of Díaz-Marsa et al., which demonstrate that impulsivity predicts self-injurious behavior in BPD and ED populations, highlight the role of behavioral dimensions in psychiatric outcomes, suggesting a potential interplay between trauma-related predictors and behavioral traits in post-TBI psychiatric disorders (28). In our multivariate analysis, the presence of intracranial hematomas did not emerge as an independent predictor of post-traumatic psychiatric disorders, which contrasts with previous studies that identified intracranial hematomas as risk factors for adverse psychiatric outcomes. This discrepancy may result from sample size limitations or the influence of unmeasured confounding factors.

Research on targeted prevention of psychiatric disorders following TBI has yielded mixed results. While some studies suggest that multi-session cognitive behavioral therapy interventions may be effective in reducing symptoms (29). Some report promising outcomes with selective serotonin reuptake inhibitors (30). Rehabilitation therapies have shown promise in reducing the risk of psychiatric disorders following TBI (31, 32). Future research should take into account individual risk factors and timing of onset of different psychiatric disorders, with a focus on developing targeted prevention interventions (18).

The study is not without limitations. Many of the included variables were subjective measures reliant on self-reported data, which may introduce bias. Future research should focus on longitudinal studies that track TBI patients over extended periods to better understand the long-term trajectories of psychiatric disorders and the factors influencing recovery. Additionally, investigations should evaluate the effectiveness of various interventions designed to improve mental health outcomes in TBI patients.

## 5 Conclusion

Unemployment, low GCS score on admission, limb injury, post-traumatic coma, frontal lobe injury, onset of psychiatric symptoms during hospitalization was identified as independent predictors of post-traumatic psychiatric disorders. Routine mental health screenings for conditions such as depression and anxiety should be integrated into the care of TBI patients.

## Data Availability

The original contributions presented in the study are included in the article/supplementary material. Further inquiries can be directed to the corresponding author.
